# Research on an Online Monitoring Device for the Powder Laying Process of Laser Powder Bed Fusion

**DOI:** 10.3390/mi15010097

**Published:** 2024-01-03

**Authors:** Bin Wei, Jiaqi Liu, Jie Li, Zigeng Zhao, Yang Liu, Guang Yang, Lijian Liu, Hongjie Chang

**Affiliations:** 1 College of Mechanical Engineering, Hebei University of Science and Technology, Shijiazhuang 050000, China; 2 Shijiazhuang Information Engineering Vocational College, Shijiazhuang 050000, China

**Keywords:** laser powder bed fusion, powder laying monitoring, convolutional neural networks, model channel pruning

## Abstract

Improving the quality of metal additive manufacturing parts requires online monitoring of the powder bed laying procedure during laser powder bed fusion production. In this article, a visual online monitoring tool for flaws in the powder laying process is examined, and machine vision technology is applied to LPBF manufacture. A multiscale improvement and model channel pruning optimization method based on convolutional neural networks is proposed, which makes up for the deficiencies of the defect recognition method of small-scale powder laying, reduces the redundant parameters of the model, and enhances the processing speed of the model under the premise of guaranteeing the accuracy of the model. Finally, we developed an LPBF manufacturing process laying powder defect recognition algorithm. Test experiments show the performance of the method: the minimum size of the detected defects is 0.54 mm, the accuracy rate of the feedback results is 98.63%, and the single-layer laying powder detection time is 3.516 s, which can realize the effective detection and control of common laying powder defects in the additive manufacturing process, avoids the breakage of the scraper, and ensures the safe operation of the LPBF equipment.

## 1. Introduction

The LPBF manufacturing process is a complex and dynamic interaction between a high-energy laser and metal powder. It involves interactions between gas, liquid, and solid phases [1]. The quality of parts manufacturing is influenced by many factors, including material properties [2], optical path systems [3], scanning characteristics [4], geometry [5], and mechanical structure [6]. The powder laying process is one of many contributing elements, and as a significant factor impacted by material characteristics and mechanical structure, it is a critical stage in the manufacturing of LPBF components. The efficacy of powder laying has a direct impact on component production quality [7]. If the powder laying layer is uneven, i.e., if there are faults in the powder laying layer, such as missing powder, streaks, or piles of powder, the surface of the component, which will be cooled and solidified by this layer following the high-energy laser melting, will be uneven [8]. The accumulation of metal powder laying powder defects over time will also result in metallurgical flaws like spheroidization, porosity, cracks, and unmelted powder, which ultimately affects the part’s manufacturing quality and, in extreme cases, can harm the powder spreading scraper and LPBF molding machinery. In order to avoid powder laying errors that result in part molding failure or damage to LPBF equipment, it is crucial to ensure that the quality of each layer of powder laying corresponds to the manufacturing needs of the part during the part manufacturing process.

**Figure 1:**
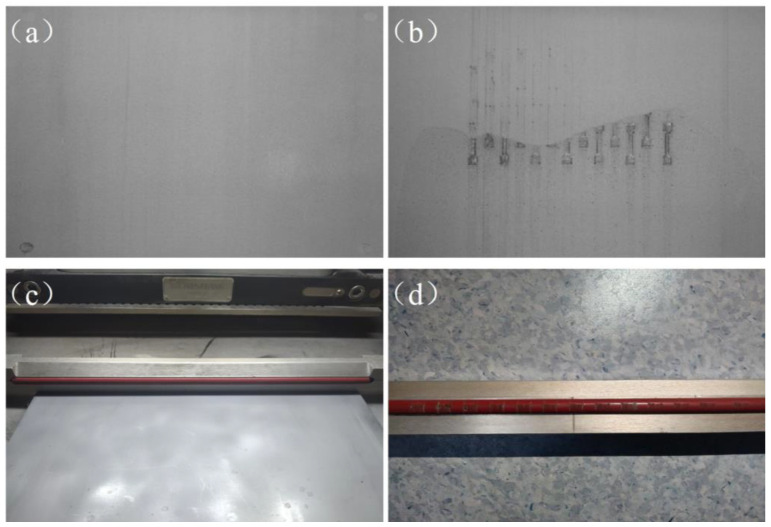
(**a**) Powder laying normal; (**b**) manufacturing failure; (**c**) squeegee tape condition before manufacturing; (**d**) squeegee tape condition after manufacturing.

In the LPBF parts manufacturing process, a squeegee performs the powder spreading process, which causes the metal powder to be spread on the molded substrate, waiting for the laser scanning process. The powder laying process is the first step in the manufacturing process to ensure that the parts are manufactured and is a critical step that has an impact on the quality of the metal LPBF manufacturing. At present, the research on the detection of powder laying quality mainly includes three stages in the molding process, which are the detection of scraper state information in the powder laying process, the detection of powder layer state after the completion of the powder laying action, and the detection of powder layer state after laser scanning and processing. In the parts manufacturing process, when the process parameters or manufacturing substrate heating temperature and other factors are not set reasonably, it will lead to parts warping, surface spheroidization, and non-fused defects, which affect the scraper spread powder, and cause serious damage to the scraper. To monitor the movement state of the scraper, B. Reinarz et al. [9] installed piezoelectric accelerometers on the powder spreading scraper, real-time monitoring of the acceleration information of the scraper in the process of powder spreading when the part exists ultra-high, the powder spreading scraper will be an ultra-high part of the existence of a certain degree of interference, resulting in different degrees of vibration, according to the speed change to analyze the protruding of the molten cladding layer. S. Kleszczynski et al. [10] continued to improve the research on this basis, the use of acceleration sensors to monitor the speed change information of the scraper laying powder and the limit sensors at both ends of the scraper operation as the start and end position of the information recording, to achieve the accurate acquisition of acceleration information at different positions. Warpage, surface spheroidization, super-high melted cladding layer, and other defects that occur during the part molding process, however, are the result of continuous accumulation. If these defects can be identified early on and appropriate action is taken to prevent the accumulation of defects, interference between the scraper and super-high part of the part can be avoided, ensuring the safe and stable operation of the LPBF manufacturing process. As a result, machine learning, machine vision, and deep learning technologies are widely used to monitor the powder layer status during LPBF molding as a way to improve part-molding quality and to avoid damage to the LPBF molding equipment. Many studies [11,12,13,14,15,16,17] have extracted defects in the powder-laying process using devices such as industrial cameras and infrared cameras coupled with image processing algorithms. For example, M. Abdelrahman [18] and others, utilized a high-resolution optical imaging monitoring system to photograph the powder bed before and after laser scanning, which used multiple light sources from different directions to construct the image, and then created a binary template from a sliced 3D model of the part, which was utilized to index the optical image data to the part geometry, which ultimately allowed for the detection of defects in the part defects in the area of the part; B. Shi et al. [19], proposed to build a powder bed inspection system using an industrial camera and multiple illumination sources, and proposed a better illumination strategy by investigating the expression of defective features under different illumination, and also utilized image feature enhancement and adaptive threshold segmentation algorithm based on the grayscale features of the powder bed image for separating defective regions and based on the three convolutional neural network algorithms, namely, AlexNet, RexNet50, and VGG16—three kinds of convolutional neural network algorithms on the current powder layer exist in the stripe, ultra-high and incomplete powder laying three types of defective regions were experimentally compared and analyzed, and the results showed that the three kinds of defective data are prone to overfitting under the complex model. Other scholars have identified and detected defects in the powder laying process by using industrial cameras, infrared cameras, thermal cameras, and other devices combined with depth algorithms [20,21,22,23,24]. The above research has realized the acquisition of scraper motion signals and powder bed images in the powder spreading process by installing piezoelectric accelerometers on the scraper, installing industrial cameras, etc. At the same time, the combination of deep learning algorithms realizes the recognition of defects in the powder spreading process, but there are still the following problems: (1) most of the studies only focus on the detection and identification of single powder laying defects, whereas the defects generated during the LPBF molding process are more complex, and multiple defects are prone to occur in a single powder laying layer; (2) most of the research is to obtain the feature information of the powder bed and scraper in the powder laying process, and then use image processing algorithms to identify, detect and analyze them, which belongs to the offline analysis, and it is not possible to realize the real-time detection of the powder laying defects in the LPBF molding process and to control the LPBF equipment to perform the suspension of the printing when the powder laying defects are serious, and other operations. A previous experimental study of the laser powder bed melting process discovered that defects in the laying of powder occurred during the printing process and staff did not detect the problem in time to deal with it in a timely manner. On the one hand, this will lead to the laying process of each layer of metal powder thickness not meeting the theoretical thickness and the manufacturing process of the metal powder over-melting or not melting, causing damage to the squeegee rubber strip. It also leads to a waste of the raw material and time costs for the metal powder. As shown in Figure 1, the JSJ100 equipment printing process, due to the laying of powder defects not being dealt with in a timely manner, then continue to accumulate, ultimately leading to the manufacturing failure.

Therefore, it is imperative to develop a powder coating quality online monitoring device with automatic real-time detection and result feedback function, which realizes real-time detection and evaluation of the quality of each layer of powder coating by visual detection technology, identifies a variety of powder coating defects, and intervenes in the manufacturing process of LPBF manufacturing equipment according to the classification results, to improve the quality of parts manufacturing and manufacturing efficiency. At the same time, recording and saving the inspection results in each layer of powder laying in the manufacturing process providing data support for subsequent parts quality tracing and process research, which is of great significance for the future development of metal additive manufacturing. In this paper, based on machine vision, deep learning, image processing, and other methods, an online monitoring device for LPBF powder laying quality is developed and deployed on the JSJ100 LPBF metal additive manufacturing equipment developed by the team. Ultimately, the accurate identification and judgment of powder laying defects are realized, and the online identification and monitoring of powder laying defects is completed, which greatly saves the material cost, time cost, and personnel cost, and at the same time ensures the safe operation of LPBF equipment.

## 2. Program Design of Online Visual Monitoring Device for the LPBF Powder Laying Process

**Figure 2:**
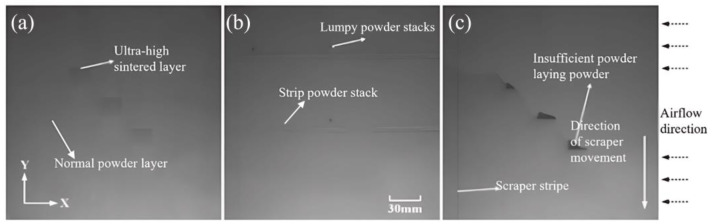
(**a**) Normal powder layer, ultra-high sintered layer; (**b**) strip powder stack, lumpy powder stacks; (**c**) insufficient powder laying powder, scraper stripe.

**Figure 3:**
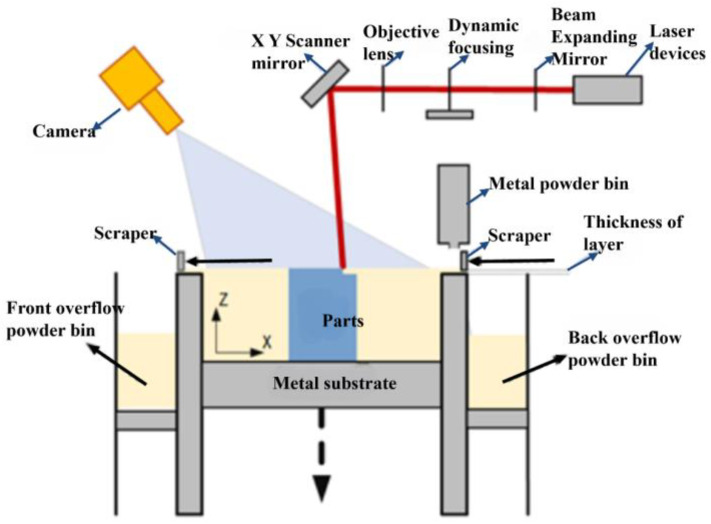
The off-axis camera mounting scheme.

The online visual monitoring device of the LPBF powder laying process is designed based on the research and development of JSJ100 equipment, which has a maximum molding size of 250 mm × 250 mm × 300 mm, and a single molding time between a few hours to dozens of hours, JSJ100 equipment process conditions parameters, as shown in Table 1. Through the preliminary experiments, it was found that the LPBF powder laying process mainly includes six powder laying states: normal, a super-high fused cladding layer, striped powder pile, a lumpy powder pile, a squeegee stripe, and an insufficient powder laying, as shown in Figure 2. The program has the following requirements that it can accurately identify: (1) the above six laying powder state defects; (2) the above laying powder defects for common size parameters of more than 1 mm, and the online monitoring system resolution of at least monitor 1 mm detail defects; (3) the equipment in the processing of medium-sized parts in a single layer of the processing time of about 2–3 min, in a single layer of laying powder time of 15–20 s, to ensure that the efficiency of the parts molding, the requirements of the system to lay a single layer of the quality of detection of the powder is less than a single layer of the time of molding the time of 5 percent, so it is necessary to control the time to detect the detection time in less than 6 s. Hardware imaging system in the overall system parameters under the same premise, when the coaxial installation of the camera when the imaging effect is the best, but taking into account the JSJ100 equipment optical circuit modification difficulties, as well as JSJ100 equipment molding bin space is small, so the use of off-axis mounting program to lay the powder process of image acquisition, off-axis camera mounting program schematic diagram in Figure 3. The software system utilizes the OPC UA protocol to achieve communication. The controller of the JSJ100 device selects the control platform of AMCP with the OPC UA server, which supports the latest OPC UA protocol, and the nodes in the server can be accessed and operated through the OPC UA client by using IDs and digital certificates. The main functions of the software design include: (1) a software communication function, mainly involved in the development of the OPC UA client function and the secondary development of the industrial camera, in which the OPC UA client function is realized using the C# language, and the OPC UA components for the development of the monitoring system software and the camera’s communication is mainly through the form of the secondary development of the camera control program embedded into the development of the software, mainly including the camera’s opening and closing, triggering, the camera’s exposure time, the image width and height parameters, and the camera mode, etc. to achieve the automation function of the software; (2) An image automatic acquisition and real-time correction function, used after it completes the powder spreading action, to pause the printing process and carry out the powder spreading to complete the variable identification. At the same time, the online monitoring software through the OPC UA subscription function realizes the identification of the variable monitoring of the off-axis installation of the camera to collect the powder spreading image as a real-time correction; (3) The detection and feedback control function of powder spreading defects, the detection and identification of defects in the collected powder spreading image and the identification of the corrected powder spreading image in the area of identification and statistics of the results, and the statistical results will be fed back to the controller of the JSJ100 equipment through the OPC UA protocol, and the equipment will execute the relevant commands of continuing to print, alarming or pausing according to the set control logic; (4) Process data storage and database functions, online monitoring system including the name of the print job, start time, the total number of layers, the image of each layer of the laying of powder, and the identification of the results of the record and save for the operator to query.

## 3. Construction of the LPBF Online Monitoring Device for the Powder Laying Process

**Figure 4:**
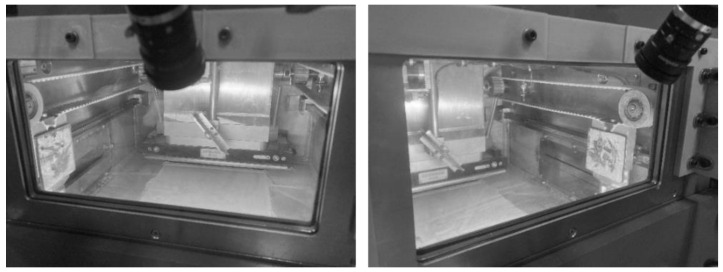
The online monitoring device for the powder spreading process.

**Figure 5:**
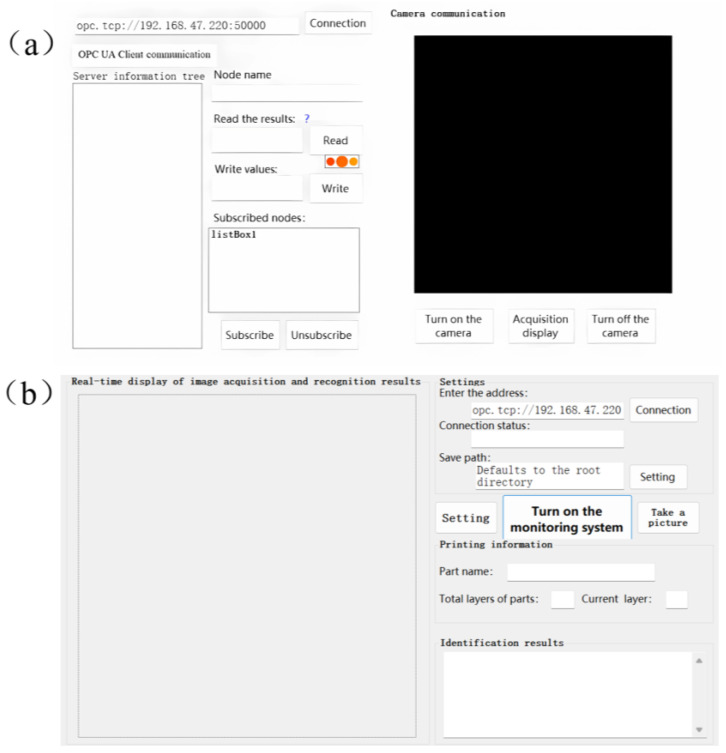
The human–computer interaction interface: (**a**) connection test interface; (**b**) monitoring system interface.

According to the online visual monitoring program design, shown in Section 2, to build the system according to the camera resolution requirements in the monitoring system, we chose Daheng Group’s industrial camera model MER-630-16GM/C-P, whose maximum resolution is 3088 (H) × 2064 (V) and frame rate is 16 fps. The selection of the lens is based on the selected industrial camera, which is known to be a type of industrial camera with a camera size of 1/1.8 inch and a target size of 7.18 mm × 5.32 mm. Due to the use of off-axis shooting, which is affected by the size of the window glass, the actual distance between the camera and the surface of the metal substrate is about 420–470 mm and taking the object distance l = 450 mm, according to the formula for calculating the focal length of the objective lens the focal length of 9.567 mm can be obtained. At the same time, taking into account the adjustment of the camera interface type, the final choice is the Japanese company Computar’s focal length of 8 mm, the model for the M0828-MPW2 lens. Taking into account the monitoring system camera is installed in the molding silo outside of the glass, when obtaining the image of the paving powder, there is an easy reflective phenomenonthat can affect the image quality, therefore, the lens is installed on the polarizer, with a light source with two perpendicular white ordinary LED lamps. The paraxial monitoring system hardware system platform is shown in Figure 4. The software system carries out the design of the human–computer interaction interface, as shown in Figure 5, and realizes the communication between the camera and the AMCP control platform.

## 4. Research on the Defect Identification Algorithm of the LPBF Powder Laying Process

### 4.1. Data Set Construction and Evaluation

#### 4.1.1. Tilted Image Correction

As the camera adopts the off-axis mounting scheme, resulting in aberration and perspective distortion in the acquired powder spread image, it is necessary to carry out camera calibration and perspective correction for this. Camera calibration uses the pinhole imaging principle to find the mathematical relationship between the points in the world coordinate system and the pixel coordinate system and completes the data conversion between the two. In this paper, we use a Halcon calibration assistant and a 7 × 7 dot calibration board to solve the parameters of the acquired 20 calibration images; the calibration results are shown in Table 2, and the image distortion correction is completed according to the obtained camera parameters.

#### 4.1.2. Perspective Correction

The perspective transformation correction of the original metal powder laying image actually maps the value of each pixel on the original metal powder laying image to the new plane in turn, with the principle equation:

(1) $$[x^{\prime }\;y^{\prime }\;w^{\prime }]=[u\;v\;w]\cdot H,$$

where (*u*, *v*) are the coordinates of each pixel point in the tilted image of the original metal powder laying powder, the parameter *w* = 1, and *H* is the transformation matrix required for calibration, which is converted to chi-square coordinates and matrices in the form:

(2) $$[x^{\prime }\;y^{\prime }\;w^{\prime }]=[u\;v\;w]\;\left[\begin{array}{l} h_{11}\;h_{12}\;h_{13}\\ h_{21}\;h_{22}\;h_{23}\\ h_{31}\;h_{32}\;h_{33} \end{array}\right].$$

In the transformation matrix *H* (*h*_11_, *h*_12_, *h*_21_, *h*_22_) denote linear transformations, (h_31_, h_32_) denote translation transformations, and (*h*_13_, *h*_23_) denote perspective transformations. We obtain the *H* matrix using the hom_vector_to_proj_hom_mat2d (: : Px, Py, Pw, Qx, Qy, Qw, method: *H*) operator.

The key parameter (Px, Py) is the set of coordinates of each point in the image before correction, and (Qx, Qy) is the set of coordinates of the corresponding points after correction, in which the coordinates of a certain point are set to be (*x*, *y*), which is normalized by making *h*_33_ = 1:

(3) $$x=\frac{x^{\prime }}{w^{\prime }}=\frac{h_{11}u+h_{21}v+h_{31}}{h_{13}u+h_{23}v+1}’$$

(4) $$y=\frac{y^{\prime }}{w^{\prime }}=\frac{h_{12}u+h_{22}v+h_{32}}{h_{13}u+h_{23}v+1}$$

**Figure 6:**
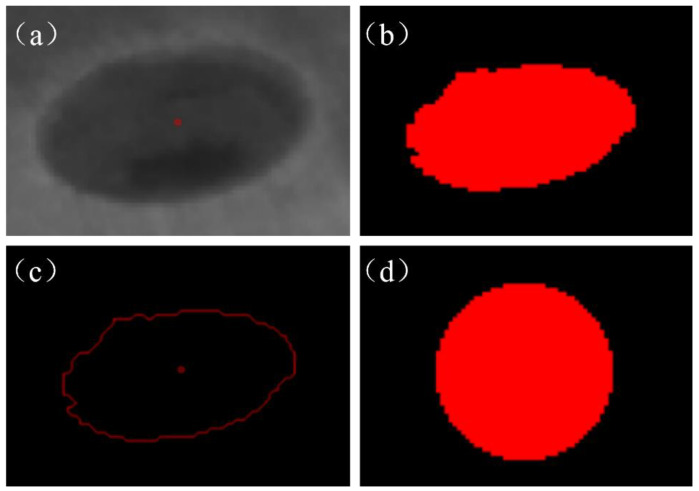
The process for extracting the coordinates of the center point of the mounting holes for manufacturing substrates.

**Figure 7:**
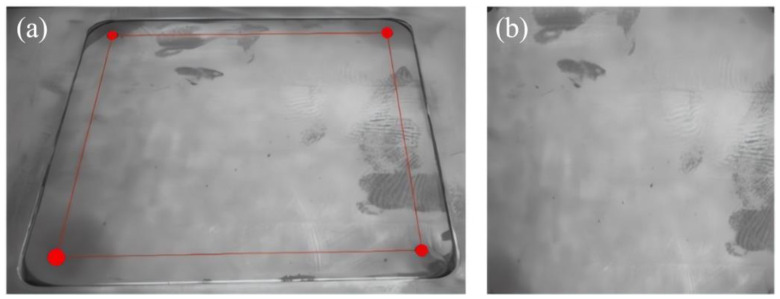
Corrected images: (**a**) actual camera image; (**b**) final part manufacturing area.

According to Formulas (3) and (4), it can be seen that there are a total of eight unknown parameters, and by a point corresponding to the *x*, *y* coordinates, it can be obtained from the two solution equations, so only four points can be solved for the *H* matrix. To obtain the perspective transformation matrix, this paper chooses to manufacture the substrate with four mounting holes in the center of the coordinates of the solution. As shown in Figure 6, the process of extracting the coordinates of the center point of the substrate mounting holes is as follows: (a) draw the ROI region, which contains an image of a substrate mounting hole; (b) process the drawn image of the ROI region by using an adaptive segmentation algorithm, and obtain the region of the mounting holes; (c) carry out the extraction of the contour of the region of the mounting holes; and (d) fit a circle on the basis of the contour of the region, and extract the coordinates of the circle’s center as the center coordinate of the position of the mounting holes. The perspective transformation matrix can be obtained by substituting the coordinates of the four points extracted by the above method into (3) and (4). Meanwhile, since the bilinear difference algorithm is characterized by high quality and strong continuity of pixel values, the bilinear interpolation method is used to supplement some of the missing points on the image after perspective transformation. In order to facilitate further processing of defects in the powder laying process, the region of interest, the image of the manufacturing region of the part, is obtained through image cropping. The original image and the final image of the manufacturing region are shown in Figure 7.

#### 4.1.3. Image Processing and Data Enhancement

**Figure 8:**
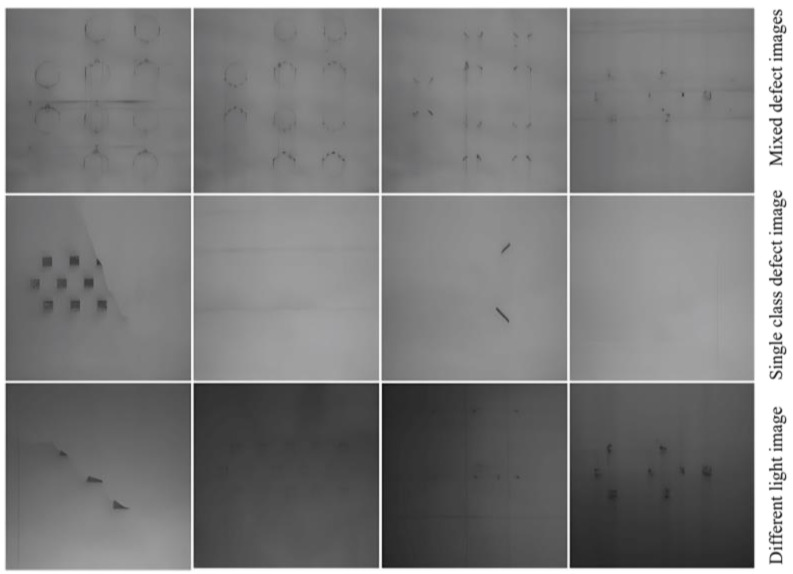
The partial metal powder spreading image.

**Figure 9:**
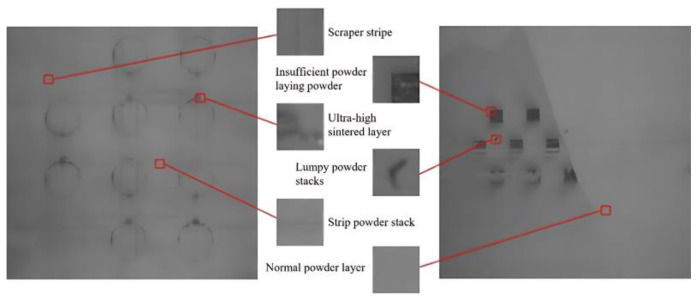
The principle of making image data of small-scale powder laying defects.

Due to the complex lighting environment in the LPBF manufacturing silo and the long manufacturing experimental period, in order to effectively enhance the diversity of the powder laying data, the powder laying images under different lighting environments and luminance were collected by interfering with the LPBF manufacturing process, respectively. Cumulatively, 1794 images of powder laying were collected through the experimental method of manufacturing molding process, including 221 effective images with common defects, and some different image data of the powder laying defects are shown in Figure 8. At the same time, considering different paving defect size characteristics, classification network recognition characteristics, and the small-scale region division recognition strategy adopted in this paper, the acquired paving images are cropped. The size of the processed powder laying defect image is 50 pixel × 50 pixel and the data annotation of the cropped defect images is performed to produce a small-scale powder laying defect dataset, and the annotation principle is shown in Figure 9. By cropping 221 valid images collected from several manufacturing experiments, a total of more than 170,000 50 pixel × 50 pixel images were obtained, but the normal image data were predominant among them, and in order to enhance the diversity of the dataset, data augmentation was used to expand the data in order to avoid the interclass imbalance phenomenon that exists in different data. Considering also that the two types of defects, the strip powder stack and scraper strips, have a fixed texture direction, three methods of contrast enhancement, rotation, and image mirroring are applied to the images to expand the dataset. The originally collected image data has been able to meet the training requirements of deep learning after the cropping process and data expansion, the dataset includes 6 categories, 2050 data images after cropping, and 5150 images have been added after data expansion, totaling 7200 images. For the normal powder layer, ultra-high sintered layer, strip powder stack, insufficient powder laying powder, and scraper stripe, there are 200 pictures of each type of metal powder laying powder state.

### 4.2. Identification of Powder Laying Defects by Small-Scale Area Division

In order to verify the effect of different class models on the extraction and recognition of features of various types of metal powder laying images, three models, AlexNet, ResNet50, and SqueezeNet, are trained and analyzed using migration learning. The environment used in this experiment is a 64-bit Windows 10 system, the processor model is Intel(R) Core(TM) i5-10400F CPU @ 2.90 GHz, and the graphics card is NVIDIA brand GeForce RTX 3060 (12 GB), loaded with the Halcon Image Processing Algorithm Library and Halcon 21.11 Deep Learning Framework, which provides thousands of image processing operators and commonly used pre-trained neural network models for image processing, training, and recognition, as well as building customized models for image classification, target detection, and image segmentation.

#### 4.2.1. Model Training

**Figure 10:**
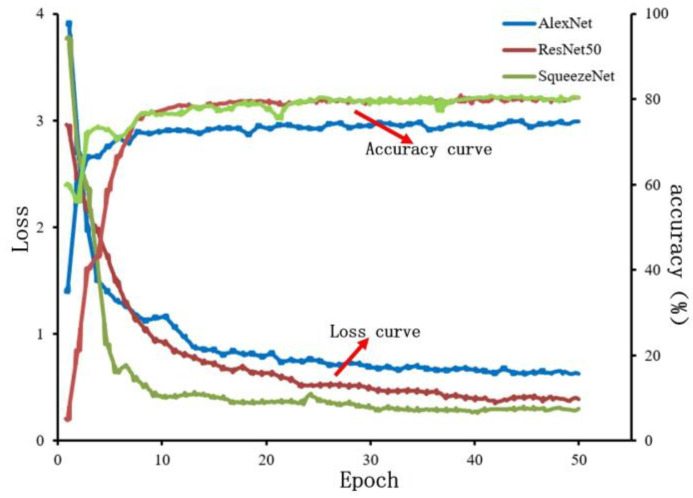
Model training results.

This experiment contains a total of 6 types of data for normal and different defective images, with a total of 7200 pieces of known training data, which are randomly divided into training, validation, and test sets in the ratio of 6:2:24,320 pieces that are used for model training, and 1440 pieces of each that are used for validation and evaluation of the model. The CNN model is trained using the momentum-based SGD optimization algorithm with hyper-parameters set as follows: the momentumwas 0.9, the learning rate was 0.001, the iteration number epoch was 50, the batch size for training was 32, and the random number seeds were set at the same time. The training results are shown in Figure 10. From Figure 10, the training loss curves show that the loss values of the three models can be stabilized after 30 rounds of training, in which the SqueezeNet model converges faster and the ResNet50 model converges slower, and from Figure 10 the accuracy curves showed that the AlexNet model had lower accuracy, and the ResNet50 and SqueezeNet models had higher accuracies, and finally the training was completed. The final size of AlexNet, ResNet50, and SqueezeNet models were 837 MB, 180 MB, and 5.63 MB, respectively.

#### 4.2.2. Evaluation of the Model

**Figure 11:**
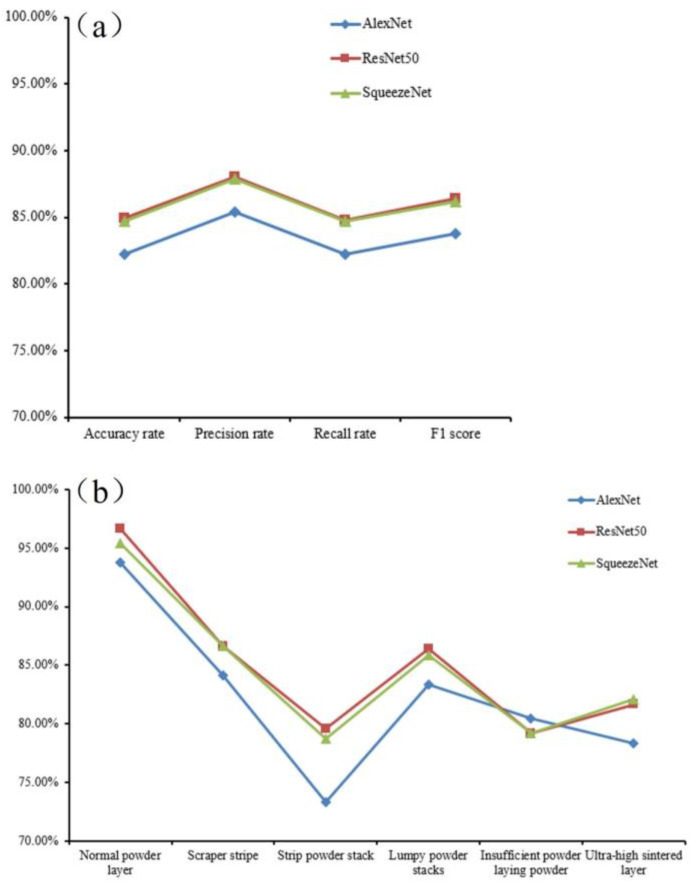
(**a**) Evaluation results of various models; (**b**) recognition accuracy of different models for each defect category in the test set.

In order to judge the accuracy of the training model, it needs to be evaluated. According to the actual needs of the classification scenarios in this paper, the accuracy, precision, recall, and F1 score are used as the evaluation indexes of the models’ effect on the recognition of laying powder defects. Another 20% of the test set data was used to evaluate the models, and the results are shown in Figure 11a. The evaluation and assessment results showed that the three models had more than an 80% recognition accuracy on the test data set, and the performance was similar to the accuracy on the validation set. The AlexNet model performed poorly, and the ResNet50 and SqueezeNet models performed better and were closer to each other. The recognition of each metal powder laying powder defect category image on AlexNet, ResNet50, and SqueezeNet models were also exported to analyze the recognition ability of different models for each category of defect images. As shown in Figure 11b, from the table, it can be seen that the three models of AlexNet, ResNet50, and SqueezeNet had more than a 70% recognition rate on each category of the defect data, and it can be seen from the analysis that the two models, ResNet50 and SqueezeNet, had better recognition results on the validation set, and the overall difference in the recognition rate was smaller.

#### 4.2.3. Heat Map Visualization and Analysis

**Figure 12:**
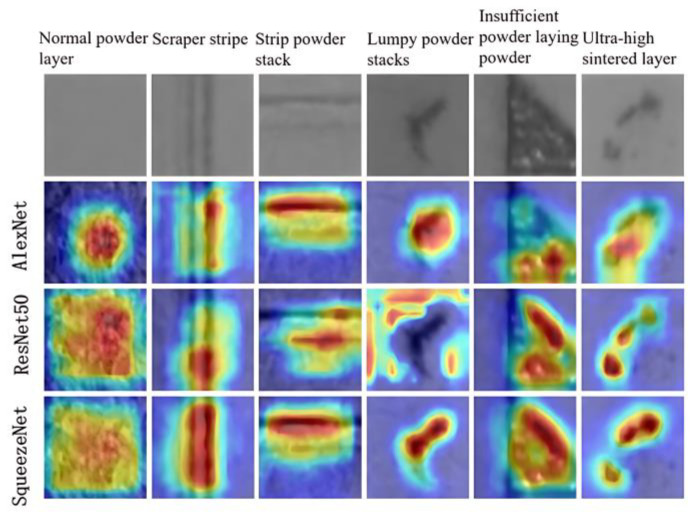
The heat map visualization of different models.

In order to more intuitively understand and analyze the identification of the above three models for various types of powder laying defects, this paper utilizes the Gradient-weighted Class Activation Mapping (Grad-CAM) technique to obtain a visual interpretation of the heat map of some of the images from the deep network through gradient-based localization, in order to analyze the distinguishing ability of the three models for the image features of different powder laying defects, as well as the image regions that are the main focus of attention when performing inference. As shown in Figure 12, the heat map visualization results of different powder laying images in the three models of AlexNet, ResNet50, and SqueezeNet are shown. Among them, the red and yellow regions indicate the regions of interest for different categories of dusted images for inference under different models. It can be observed that among the three trained models, the SqueezeNet model was more accurate in focusing on the region of interest when performing image inference, which is beneficial in accurately performing inference on the powder laying defects.

### 4.3. SqueezeNet Model-Based Multiscale Improved Method for Identifying Powder Laying Defects

Through the comparative experimental analysis of the three models in Section 4.2, it can be seen that the SqueezeNet model had better overall performance in order to further improve the accuracy and recognition efficiency of the paving defects in a comprehensive way, this section mainly focuses on the improvement and optimization of the small-scale paving defect recognition method based on the SqueezeNet model. A multiscale method is proposed to analyze the deficiencies in the recognition of defects in the small-scale pavement images, and a multiscale pavement dataset is constructed for the method, followed by model training, evaluation, and feature map visualization.

#### 4.3.1. Multiscale Powder Laying Defect Identification Methods

**Figure 13:**
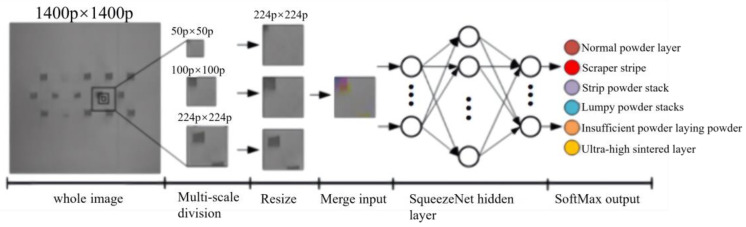
The principle of recognizing multiscale powder laying defects.

The incorrectly identified powder laying image in Section 4.2 was analyzed, and it was found that the main reason was due to the fact that part of the image contained only the edge information of the defects during the 50 pixel × 50 pixel division, which resulted in the unclear feature class of the defects represented in this image. Considering that the small-scale pavement defect dataset will be consciously labeled according to the pavement state of the surrounding area when performing the production of the small-scale pavement defect dataset, the introduction of the influence of the pavement state of the surrounding area on the recognition results when performing the training and recognition of the original 50 pixel × 50 pixel defect image can improve the shortcomings in recognition of the small-scale pavement defects. In view of the analysis results and the CNN’s requirements on the input data, a multiscale powder laying defect recognition method was proposed, which mainly combined the original 50 pixel × 50 pixel region, the 100 pixel × 100 pixel, and 224 pixel × 224 pixel powder laying images centered on the region into a three-channel image, and keeping the original defect labels unchanged, as the new model training set. It added the ability to perceive the powdering state of the surrounding area in the model training and classification process to improve the shortcomings that exist when using only small-scale powdering image recognition, the principle of which is shown in Figure 13.

#### 4.3.2. Data Set Construction

**Figure 14:**
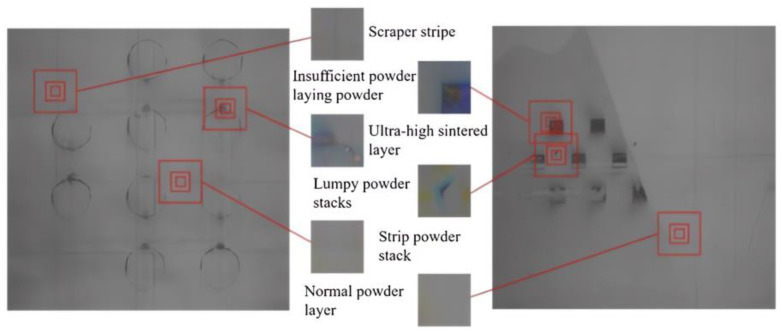
Principle of creating a multiscale image dataset of powder laying defects.

The construction of the powder-laying defect dataset was carried out according to the proposed multiscale powder-laying defect identification method. The construction of the multiscale dataset was based on the whole LPBF molding process powder laying defect image corrected in Section 4.1, and the original dataset and its surrounding areas were cropped and channel-merged. The production principle is shown in Figure 14. Then the image processing methods such as contrast enhancement, rotation, and mirroring with the same parameters, as discussedin Section 4.1, were used to enhance the powder laying image data.

#### 4.3.3. Model Training

**Figure 15:**
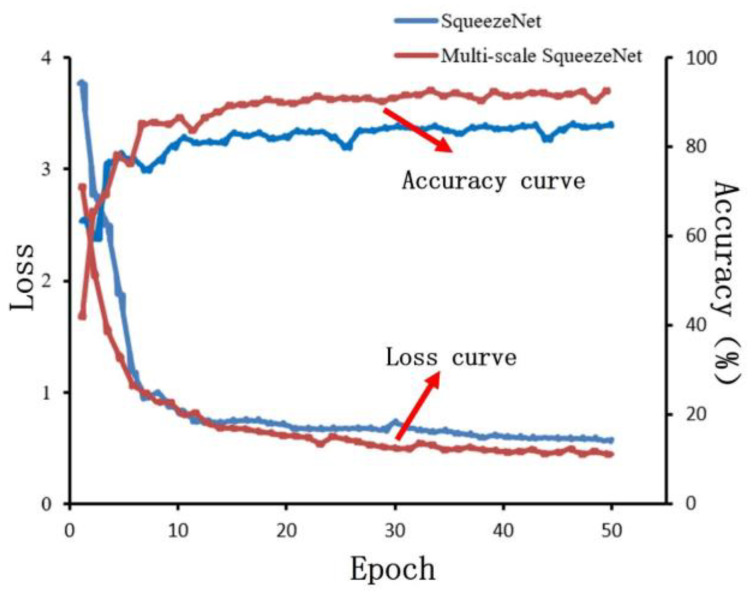
Model training results.

The CNN model was trained using the momentum-based SGD optimization algorithm, in which the hyperparameters were set as follows: the momentum was 0.9, the learning rate was 0.001, the iteration number Epoch was 50, the batch size of the training Batch Size was 32, and at the same time set the random number of seeds. The data of the training process is shown in Figure 15, which shows that the multiscale SqueezeNet model performs well on the multiscale powdered image dataset, and the loss value tends to be 0.5 at 30 rounds of iteration. It can be seen that the multiscale SqueezeNet model performs better on the multiscale pavement image dataset, and from the loss function curve, the model tends to 0.5 loss value in 30 rounds of iteration and remains stable in the subsequent iteration process. Similar to the change rule of the loss curve, the accuracy curve of the model tended to stabilize after 30 rounds of iteration.

#### 4.3.4. Model Evaluation

**Figure 16:**
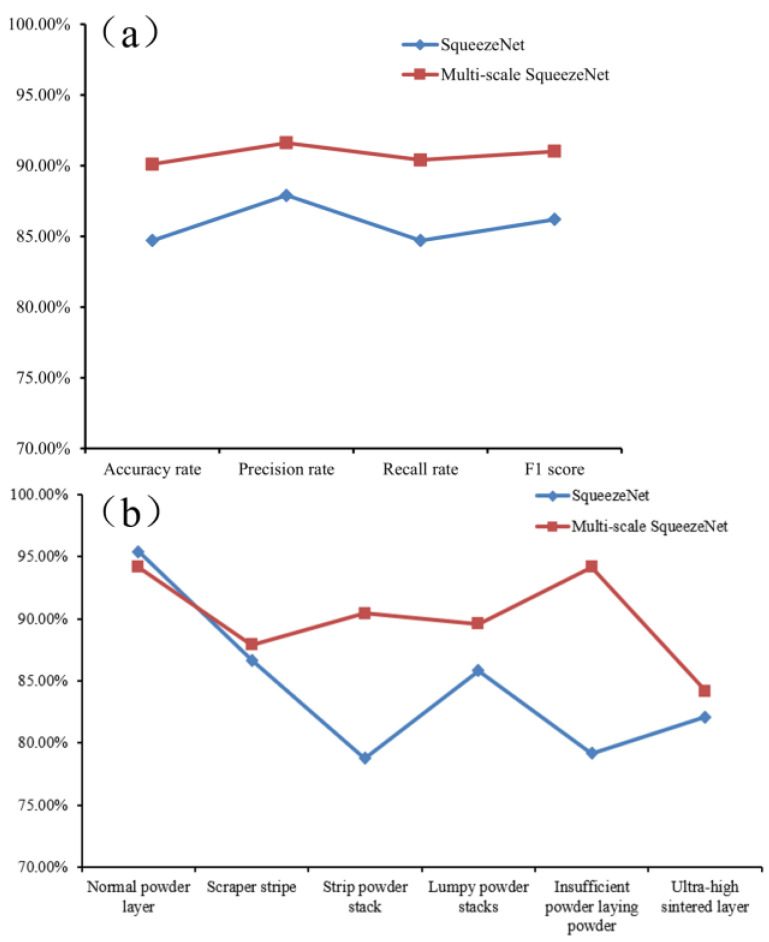
Model evaluation results.

The model needs to be evaluated at the end of model training to determine the training quality of the model. The model was evaluated using another 20% of the test set data, which had a total of 1440 images of six categories, including normal, squeegee stripe, bar powder accumulation, block powder accumulation, underlayment, and fused cladding layer ultra-high. The results are shown in Figure 16. From the figure, it can be seen that the accuracy performance of the SqueezeNet model based on multiscale improvement was better, and all evaluation indexes were improved. Its accuracy, precision, recall, and F1 score increased compared to before the improvement, as shown in Figure 16a, and the recognition rate of each powder laying defect category in the test set was also increased, as shown in Figure 16b.

#### 4.3.5. Feature Map Visualization

**Figure 17:**
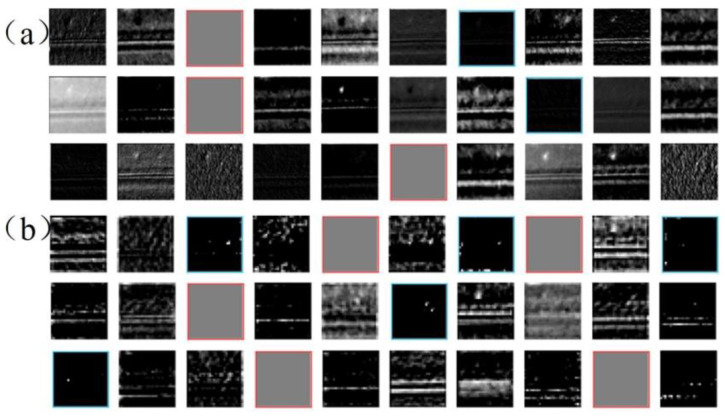
(**a**) Partial feature map visualization of the Conv1 layer; (**b**) feature map visualization of the output layer of the fifth Fire module.

In order to better observe the learning of the improved multiscale SqueezeNet model on the powder defect images, the feature channels of the first convolutional layer “Conv1” and the fifth Fire module output layer “Fire5_concat” in the recognition process of the powder defect images were selected and visualized [25]. The first convolutional product layer had a total of 64 feature channels, and some of the channels were visualized, as shown in Figure 17a. The fifth Fire module had a total merged output of 256 feature channels, and again, some of the channels were selected for visualization, as shown in Figure 17b. Different convolutional kernels differently characterized the multiscale SqueezeNet model during forward propagation and some deep convolutional kernels were useless for normal powdered areas in the defective image, which was considered because the normal powdered image area cannot be used as an effective feature extraction area for distinguishing between the six classes of images. As the convolutional layers deepened, the features learned by the model became more abstract and visually uninterpretable, due to the fact that deeper layers showed less visual information and more abstract information related to image categories. At the same time, the sparsity of feature activation increased with the deepening of the convolutional layer, such as the visualization results of the red border feature image indicate that the corresponding channel is not activated, which is due to the fact that the feature pattern encoded by this channel was not found in the input image, and the image features extracted from the feature channel corresponding to the blue border had less influence on the final decision of this pavement image. Therefore, it is concluded that some of the channels in the SqueezeNet model trained based on the multiscale pavement image dataset were not activated when performing image inference, or the extracted features have less influence on the final decision, and there is a certain amount of parameter redundancy, which can be removed by pruning techniques to obtain a compact, less complex, and a more targeted multiscale pavement defect recognition model.

### 4.4. Channel Pruning Model Optimization Method

Through the visual analysis of the feature channels of the multiscale SqueezeNet model, it can be seen that some of the channels in the SqueezeNet model trained on the multiscale pavement image dataset were not activated when performing image inference, or the extracted features had less influence on the final decision, and there was a certain amount of parameter redundancy, so the model optimization method of channel pruning is proposed to remove them, and to reduce the redundant parameters of the model to enhance the speed of the model under the premise of guaranteeing the accuracy of the model. In order to prevent excessive pruning resulting in model performance degradation, this paper adopts an iterative pruning strategy, i.e., removing part of the proportional convolution kernel each time, realizing the pruning goal through multiple iterations, and counting the changing relationship between the pruning rate and the various model performances.

#### Analysis of Pruning Results

(1) Changing patterns of model accuracy and storage space at different levels of pruning.

**Figure 18:**
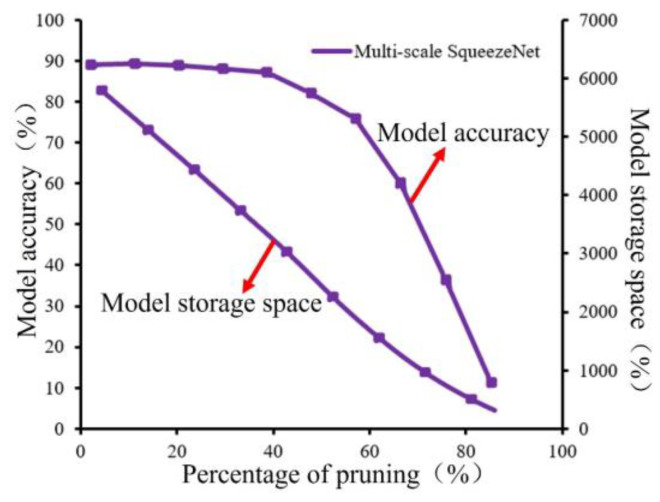
Relationship between the model accuracy and model storage space and percentage of purning.

The variation of model accuracy and storage space in relation to different degrees of pruning is shown in Figure 18, from which it can be analyzed that as the percentage of pruning increases, both model accuracy and model size show an overall decreasing trend. Among them, the model size decreases steadily with it, which is approximately linearly correlated. The model accuracy changes less when the pruning percentage is less than 40% or less, and the accuracy loss is within 1% and shows an accelerated decreasing trend when the pruning percentage is more than 40%. According to the data analysis, it can be concluded that when the pruning percentage is 40% or less, the multiscale SqueezeNet model pruning based on Oracle pruning standard can reduce the model channel redundancy under the premise of guaranteeing the model accuracy, so the multiscale SqueezeNet model with a pruning percentage of 40% is considered as the optimal model for monitoring powder laying defects of metal powders in this paper, MC-SqueezeNet.

(2) Changes in reasoning speed before and after model pruning.

**Figure 19:**
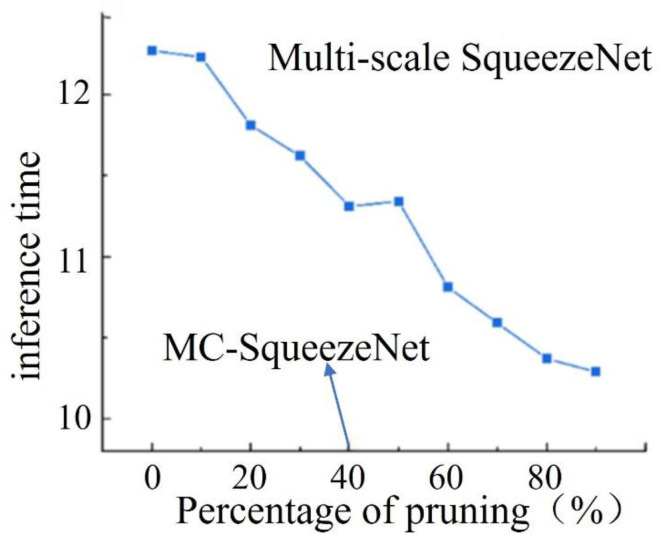
Model inference time versus different levels of pruning.

Through inference experiments on the model after pruning at each stage, it is concluded that traditional convolutional neural network can improve the inference speed of the model to a certain extent after channel pruning. As Figure 19 shows, the relationship between the inference speed of the multiscale SqueezeNet model and the percentage of pruning, where the inference time was the average of the time required for the model to perform inference on 5000 multiscale images of powder laying defects five times. From the figure, it can be seen that the initial network for the 5000 multiscale paving powder defect image prediction time consumed 12.27 s, with the increase of the total pruning percentage of the model, the recognition model inference time shows a general trend of reduction, when the percentage of pruning was greater than 10%, the inference time began to have a significant decrease, and it can be seen that the image inference time of the MC-SqueezeNet model was 11.31 s, compared to the 7.8% reduction before pruning.

(3) Change in the number of convolution kernels in each layer of the model before and after pruning.

The changes in the number of convolutional kernels in each layer of the multiscale SqueezeNet model when the pruning percentage is 40% are shown in Table 3. The number of convolutional kernels in the Fire module was combined, and the table shows that the percentage of convolutional kernel removal shows an overall increasing trend with the increase of network depth, which is also consistent with the analysis results of the feature map visualization.

## 5. Experimental Validation of Defect Identification Algorithms for Metal Powder Spreading Process

### 5.1. Experimental Equipment and Materials

**Figure 20:**
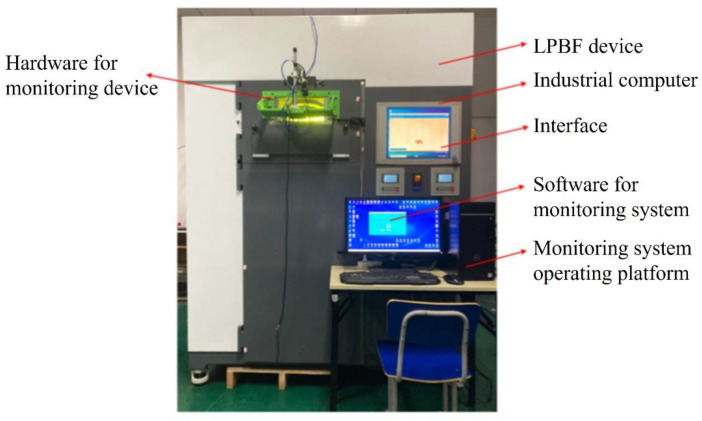
JSJ100 LPBF equipment and monitoring device.

The experimental equipment is the JSJ100 LPBF molding equipment mentioned in Section 3 and the online monitoring device designed and developed in this paper, of which the structure of each part is shown in Figure 20.

For this experiment, Renishaw 316L stainless steel powder was used as the raw material, and the particle size of the powder was between 15–45 μm. Before conducting the experiment, the impurities were first screened out using a sieving machine and dried at 200 °C for 2 h. At the same time, in the molding experiment process the molding bin provided an argon gas environment to prevent high-temperature oxidation on the parts manufacturing process.

### 5.2. Manufacturing Experiment and Analysis

Through a specific part of the manufacturing experiment for this paper LPBF molding metal powder laying powder quality online monitoring device verification, for the part a total of two printing experiments, in order to obtain the experimental effect, the experimental process will be monitoring the system’s feedback control function is set to pop-up window prompts, by the manual judgment of whether to implement the feedback control, experiments set the detection of various types of defects threshold of 3, that is, the identification of the results of a certain type of defects in the number of 3 and above, recognized as a valid category of defects in the detection of the results.

(1) The first experiment

**Figure 21:**
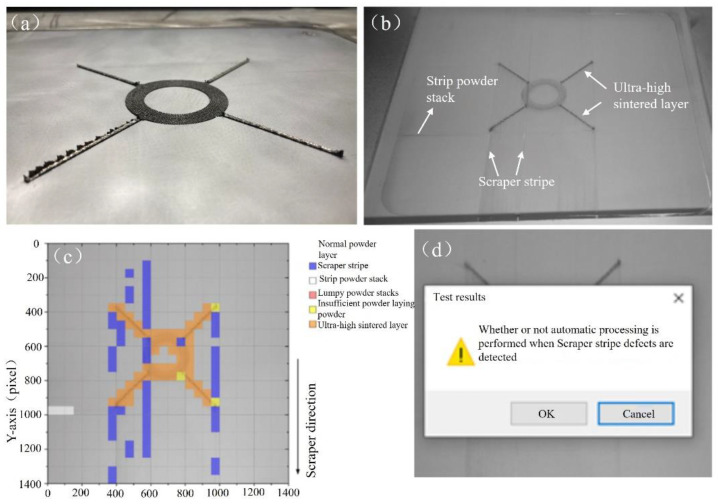
(**a**) Part fabrication results; (**b**) the 58th layer of metal powder laying image; (**c**) the 58th layer of laying image defect recognition effect; (**d**) the 58th layer of laying image inspection results.

In order to obtain the laying powder defects and to verify this paper’s online monitoring system defect detection and feedback function to meet the requirements set, we conducted the first manufacturing experiment, with the results shown in Figure 21a. It can be clearly seen that there was a serious phenomenon of the part being too high, and when the 59th layer of the laying powder action is too high, as well as the interference with the squeegee, therefore the experiments were stopped. The 58th layer of powder image is shown in Figure 21b. The manufactured part shows an area of ultra-high area damage to the scraper adhesive strip, so that the process of spreading the powder produced with its side of the movement of the scraper stripe caused defects, ultra-high serious areas, and the scraper body collision jitter, resulting in defects perpendicular to the direction of its movement of the collision stripe.

(2) Second manufacturing experiment.

**Figure 22:**
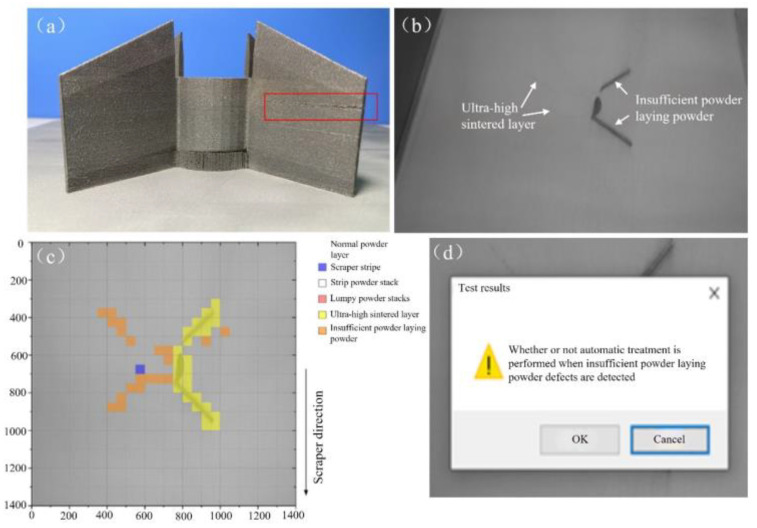
(**a**) Part fabrication results; (**b**) the 654th layer of metal powder laying image; (**c**) the 654th layer of laying image defect recognition effect; (**d**) the 654th layer of laying image inspection results.

In order to verify the stability of the online monitoring system in this paper over a long period of time, the original 316L process parameter package was invoked during the second molding experiment to finalize the part fabrication with a total duration of more than 20 h. Insufficient powder during the manufacturing process led to the 648th to 657th layer of the right side of the region There was insufficient powder spreading defects. The part molding results are shown in Figure 22a, where it can be clearly seen that there is a gap in the red box area, corresponding to the 654 layers of the powder image state shown in Figure 22b. As shown in Figure 22c, it shows the recognition effect of the 654th layer of powder state and in Figure 22d, for the layer of powder detection results.

(3) Online detection accuracy and detection time.

In order to verify whether the detection accuracy and detection speed of the single-layer powder laying quality of the online monitoring device meets the requirements of the system, the feedback results of the identification of each layer of powder laying and the time-consuming detection of defects in the experimental process are statistically and analytically analyzed. Table 4 shows the above experimental process for each layer of powder laying quality of the feedback results and the real powder laying situation of the comparison results. As can be seen from the table, in the above two experiments, for a total of 1460 layers of powder laying quality detection, the system’s feedback accuracy was 98.63%. The analysis of the wrongly identified layers was mainly due to the fact that various types of defects were not obvious at the initial stage, and the number of certain types of defects detected did not exceed the set threshold value of 3, which were not recorded as valid defects.

Table 5 shows the time-consuming data for three different stages of the online inspection of the quality of 50 layers of powder laying. The average time consumed in the three stages of image acquisition, tilt correction and storage, and partition and identification was 0.795 s, 0.159 s, and 2.562 s, totaling 3.516 s, meeting the time-consuming requirements for testing set in Section 2. After the above two manufacturing experiments and recognition effect analysis, it can be seen that by setting various types of defect thresholds, we can effectively avoid the influence of part of the powder laying area misdetection on the recognition results of the whole layer. When the threshold value of all kinds of defects was set to 3, the feedback accuracy rate of the two molding experiments was 98.63% for the cumulative quality of 1460 layers of paving powder, among which the average time consumed in the online detection of the quality of 50 layers of paving powder was 3.516 s. At the same time, the monitoring system did not show any abnormality when it worked continuously for more than 20 h and recognized the paving powder image of 1402 layers and the multiscale paving powder block for more than 1,090,000 times, which is characterized by a good recognition stability.

## 6. Conclusions

Through the study of the metal powder laying state in the LPBF molding process, an online monitoring device for the laying process was built, and the algorithm for identifying the laying defects was developed, and finally experimental validation was carried out, and better results were obtained. It is shown that this online monitoring system for laying powder is suitable for both simple and complex parts. The effective identification of powder laying defects by this monitoring device has the following significance: on the one hand, it reduces the manufacturing defects of the parts and improves the manufacturing quality and mechanical properties of the parts; on the other hand, it avoids the damage of the scraper and ensures the safe operation of the LPBF equipment, which greatly saves the time cost and labor cost. The specific conclusions of this paper are as follows.

We propose a recognition method of small-scale regional division of powder laying defects, the division of the image size of 50 pixel × 50 pixel, the construction of a small-scale powder laying defects dataset for the method, and the experiments and analyses of three different complexity models, namely, AlexNet, ResNet50, and SqueezeNet, have been completed. The results show that the method can be used for the detection of common powder laying defects, in which the SqueezeNet model had the best performance.

Aiming at the shortcomings of the small-scale powder laying defect detection method, a multiscale improvement method based on SqueezeNet model is proposed. The original small-scale region and the 100 pixel × 100 pixel and 224 pixel × 224 pixel powder laying images centered on the region were combined into a three-channel image, which was used as a multiscale dataset for the model training in order to increase the model’s ability to perceive the powder laying state around the original small-scale region. The results show that the method improved the recognition accuracy of three types of defects, namely, lumpy power stacks, insufficient powder laying power, and ultra-high fusion cladding layers.

For the parameter redundancy problem of the multiscale SqueezeNet model, an iterative pruning method is proposed to prune the model channels under the premise of guaranteeing the accuracy of the model, and better results are obtained.

The deployment of MC-SqueezeNet model and the development of online monitoring device system software were completed using OPC UA development components and .Net Framework platform, and experimental verification was conducted. The results show that the system can recognize the minimum size of defects is 0.54 mm, the accuracy of the feedback results is 98.63%, the recognition speed is 3.516 s, and it works online for more than 20 h, and all the indexes meet the design requirements.

## Figures and Tables

**Table 1:** The JSJ100 equipment process conditions parameters.

Laser spot diameter	0.07 mm
Laser power	0–400 W
Laser scanning speed	0–5000 mm/s
Layer thickness	0.05–0.1 mm
Inert protective gas	Argon gas

**Table 2:** Camera position.

X	−149.196 mm	X-direction rotation	322.433°
Y	−129.95 mm	Y-direction rotation	358.278°
Z	481.991 mm	Z-direction rotation	355.92°

**Table 3:** Changes in the number of convolution kernels in each layer before and after optimization.

Hierarchy	Number of Convolution Kernel		Removal Ratio
	Multiscale SqueezeNet	MC-SqueezeNet	
Conv1	64	62	3%
Fire2	144	124	14%
Fire3	144	127	12%
Fire4	288	222	23%
Fire5	288	219	24%
Fire6	432	243	44%
Fire7	432	258	40%
Fire8	576	290	50%
Fire9	576	251	56%
Conv10	6	6	0%

**Table 4:** Online inspection results of metal powder laying quality.

The First Print Experiment			The Second Printing Experiment			System Accuracy
Total Number of Layers	Identify the Correct Number of Layers	Accuracy Rate	Total Number of Layers	Identify the Correct Number of Layers	Accuracy Rate	
58	54	93.1%	1402	1386	98.89%	98.63%

**Table 5:** Time-consuming data for different detection stages.

Phase	Image Acquisition	Tilt Correction and Storage	Partition and Identification	Total
Average time consuming (s)	0.795	0.159	2.562	3.516

## Data Availability

All data needed to evaluate the conclusions of this manuscript are present in the paper.
